# Hydrogen Peroxide Causes Cell Death via Increased Transcription of HOXB13 in Human Lung Epithelial A549 Cells

**DOI:** 10.3390/toxics8040078

**Published:** 2020-09-28

**Authors:** Naoki Endo, Takashi Toyama, Akira Naganuma, Yoshiro Saito, Gi-Wook Hwang

**Affiliations:** 1 Laboratory of Molecular and Biochemical Toxicology, Graduate School of Pharmaceutical Sciences, Tohoku University, 6-3 Aoba, Aramaki, Aoba-ku, Sendai, Miyagi 980-8578, Japan; naoki.endou@mb.kyorin-pharm.co.jp (N.E.); takashi.toyama.c6@tohoku.ac.jp (T.T.); naganuma@tohoku.ac.jp (A.N.); 2 Laboratory of Molecular Biology and Metabolism, Graduate School of Pharmaceutical Sciences, Tohoku University, 6-3 Aoba, Aramaki, Aoba-ku, Sendai, Miyagi 980-8578, Japan; yoshiro.saito.a8@tohoku.ac.jp; 3 Watarase Research Center, Kyorin Pharmaceutical Co., Ltd., 1848, Nogi, Nogi-machi, Shimotsuga-gun, Tochigi 329-0014, Japan; 4 Laboratory of Environmental and Health Sciences, Faculty of Pharmaceutical Sciences, Tohoku Medical and Pharmaceutical University, 4-4-1 Komatsushima, Aoba-ku, Sendai, Miyagi 981-8558, Japan

**Keywords:** HOXB13, hydrogen peroxide, oxidative stress, reactive oxygen species (ROS)

## Abstract

Although homeobox protein B13 (HOXB13) is an oncogenic transcription factor, its role in stress response has rarely been examined. We previously reported that knockdown of HOXB13 reduces the cytotoxicity caused by various oxidative stress inducers. Here, we studied the role of HOXB13 in cytotoxicity caused by hydrogen peroxide in human lung epithelial A549 cells. The knockdown of HOXB13 reduced hydrogen peroxide-induced cytotoxicity; however, this phenomenon was largely absent in the presence of antioxidants (Trolox or *N*-acetyl cysteine (NAC)). This suggests that HOXB13 may be involved in the cytotoxicity caused by hydrogen peroxide via the production of reactive oxygen species (ROS). Hydrogen peroxide also increased both the mRNA and protein levels of HOXB13. However, these increases were rarely observed in the presence of a transcriptional inhibitor, which suggests that hydrogen peroxide increases protein levels via increased transcription of HOXB13. Furthermore, cell death occurred in A549 cells that highly expressed HOXB13. However, this cell death was mostly inhibited by treatment with antioxidants. Taken together, our findings indicate that HOXB13 may be a novel factor involved in the induction of oxidative stress, which causes cell death via intracellular ROS production.

## 1. Introduction

Many respiratory diseases, such as interstitial pneumonia associated with collagen disease, chronic obstructive pulmonary disease (COPD), and acute respiratory distress syndrome (ARDS), are associated with damage to lung epithelial cells, mainly caused by oxidative stress [1,2]. The lungs, an organ that exists in a highly oxygenated environment and requires a large blood supply to a large surface area, are susceptible to damage mediated by reactive oxygen species (ROS) [3]. ROS may be involved in the remodeling of extracellular matrix, apoptosis, and cell proliferation [4]. Anti-oxidative factors such as catalase, glutathione peroxidase, and superoxide dismutase are expressed in the lungs and maintain its redox states [5,6,7]. Conversely, NADPH oxidase, nitric oxide synthase, and mitochondria are sources of endogenous ROS [8]; however, the factors that are involved in promoting ROS production in the lungs have not been fully investigated.

Transcription factor homeobox protein B13 (HOXB13) is highly expressed during the developmental stage and essential for vertebrate embryonic development [9]. Although HOXB13 expression is reported to be negatively regulated by anti-sense RNA and methylation of its promoter region, reports of positive regulation of its expression are obscure [10,11]. HOXB13 is also expressed in some adult tissues and known as an oncogenic transcription factor that is associated with the malignancy of ovarian cancer, prostate cancer, and also lung adenocarcinoma [12,13,14]; however, the physiological/pathophysiological role of HOXB13 remains controversial. In a previous study, we reported that HOXB13 is involved in cell death caused by hydrogen peroxide and thiol-reactive electrophiles [15]. This indicates that HOXB13 has a novel function as a potentiator of cytotoxicity caused by oxidative stress. Therefore, in this study, we elucidate the role of HOXB13 in cell death caused by hydrogen peroxide in human lung epithelial A549 cells.

## 2. Materials and Methods

### 2.1. Cell Culture and Transfection

Human lung epithelial A549 cells were cultured in Dulbecco’s modified Eagle’s medium (DMEM) (Thermo Fisher Scientific Inc., Waltham, MA, USA), supplemented with 10% heat-inactivated fetal bovine serum (FBS) and antibiotics (100 IU/mL penicillin and 100 mg/mL streptomycin), in a humidified incubator with 5% CO_2_/95% ambient air at 37 °C. Before the experiment, 2.5 × 10^4^ cells were seeded onto a 48-well plate or 2 × 10^5^ cells were seeded onto a 6-well plate. siRNA transfection was performed using Lipofectamine RNAi MAX Transfection Reagent (Thermo Fisher Scientific Inc.), and plasmid DNA transfection was done by Lipofectamine LTX (Invitrogen, Carlsbad, CA, USA), according to the manufacturer’s instructions. Target sequence of HOXB13 siRNA (Qiagen, Hilden, Germany) was 5′-UACGCUGAUGCCUGCUGUCAA-3′ and specificities of HOXB13 siRNA and AllStars^®^ negative control siRNA (Qiagen) were predicted by the manufacturer.

### 2.2. Measurement of Cytotoxicity

A549 cells were seeded onto a 48-well plate and cultured for 24 h before exposure to hydrogen peroxide. Additionally, cytotoxicity was evaluated using *N*-acetyl cysteine (NAC) as an antioxidant against hydrogen peroxide in this study. Cells were treated with hydrogen peroxide alone or hydrogen peroxide and NAC (100 µM) or Trolox (10 µM) and cultured at 37 °C for 24 h. Cytotoxicity was evaluated based on the lactate dehydrogenase (LDH) levels in the medium using the LDH assay kit (Dojindo Laboratories, Kumamoto, Japan). After the exposure of indicated concentration of hydrogen peroxide with or without antioxidants (NAC (100 µM) or Trolox (10 µM)) for 24 h, 100 µL of medium was collected from each well and transferred to a 96-well plate. Next, the working solution was added to each well and incubated to react at 37 °C for 30 min. After the incubation, 50 μL of stop solution was added to each well. Finally, the absorbance at 490 nm was measured using an Infinite M1000 Pro (Tecan Japan Co., Ltd., Kanagawa, Japan), and changes in LDH levels in each well were calculated from the absorbance at 490 nm. Cytotoxicity was also evaluated by alamerBlue assay. After the exposure of hydrogen peroxide, the medium was changed to 90% DMEM, 10% alamerBlue solution (Invitrogen) and further incubated at 37°C for 2 h. Ex 540 nm/Em 590 nm were measured as alamerBlue value.

### 2.3. Measurement of mRNA Levels by Quantitative PCR

A549 cells were seeded onto a 6-well plate and cells were treated with hydrogen peroxide for the indicated time and at the indicated concentration. Total RNA was purified using the RNeasy plus mini kit (Qiagen) according to the manufacturer’s instructions. Complementary DNA was prepared from 500 ng of total RNA using the PrimeScript™ RT reagent kit with oligo dT primer (Takara, Shiga, Japan). Quantitative PCR (qPCR) was performed using SYBR premix Ex Taq (Takara) with a ViiA 7 real-time PCR system (Thermo Fisher Scientific). Fold changes in mRNA levels were determined, and mRNA levels were normalized to those of glyceraldehyde-3-phosphate dehydrogenase (GAPDH). Data are represented as relative mRNA expression with control as 1. Sequences of primers are as follows. HOXB13 forward; 5′-CAGATGTGTTGCCAGGGAGAAC-3′, HOXB13 reverse; 5′-AGGCGTCAGGAGGGTGCT-3′, GAPDH forward; 5′-GCACCGTCAAGGCTGAGAAC-3′, GAPDH reverse; 5′-TGGTGAAGACGCCAGTGGA-3′, catalase forward; 5′-TTTCCCAGGAAGATCCTGAC-3′, catalase reverse; 5′-ACCTTGGTGAGATCGAATGG-3′, glutathione peroxidase1 (GPx1) forward; 5′-GACTACACCCAGATGAACGAGC-3′, GPx1 reverse; 5′- CCCACCAGGAACTTCTCAAAG-3′.

### 2.4. Immunoblotting

Cells were harvested using RIPA buffer (Fujifilm-Wako, Osaka, Japan), supplemented with protease inhibitor cocktail (Roche, Indianapolis, IN, USA), according to the manufacturer’s instructions. Protein concentrations in the cell lysates were determined by DC protein assay kit (Bio-Rad, Hercules, CA, USA). Aliquots of lysates were subjected to SDS-polyacrylamide gel electrophoresis (SDS-PAGE). The resulting gel was transferred to an Immobilon-P membrane (Millipore, Burlington, MA, USA) and immunoblotted with anti-HOXB13 antibody (Cell Signaling Technologies, Danvers, MA, USA), anti-GAPDH antibody (Cell Signaling Technologies), anti-V5 tag antibody (Fujifilm-Wako), and cleaved-caspase 3 antibody (Cell Signaling Technologies). Densitometric analysis was performed by ImageJ software (https://imagej.nih.gov/ij/index.html).

### 2.5. Construction of V5 Tag Fused HOXB13 (HOXB13-V5)-Expressing Plasmid

Complementary DNA from HEK293 cells was used as a template. The coding region of the HOXB13 gene was amplified by PrimeSTAR (Takara) using the following primers: 5′-CCCAAGGTACCATGGAGCCCGGCAATTATGCCACCTTGG-3′ (sense) and 5′-GTGAAGAAC AGCGCTACCCCTGGTACCTTGGG-3′ (antisense). The resulting PCR product was ligated into the pEF5V/FRT/V5-DEST vector using Kpn1 (New England Biolabs, Ipswich, MA, USA) and Ligation high (Toyobo, Osaka, Japan) according to the manufacturer’s protocol. The plasmid was transformed into DH5α (Toyobo) for amplification and purified using the Plasmid maxi kit (Qiagen). Sequences of the above plasmids were confirmed by Sanger sequencing (Fasmac, Kanagawa, Japan).

### 2.6. Measurement of Intercellular Reactive Oxygen Species

A549 cells transfected with the HOXB13-V5 plasmid were re-seeded onto a 48-well plate and cultured for 24 h. The ROS production levels in the cells were evaluated by measuring the fluorescence intensity of 2′,7′-dichlorodihydrofluorescein (DCF), which is the deacetylated form of 2′,7′-dichlorodihydrofluorescein diacetate (DCFDA) generated by ROS in the cells. Cells were washed and culture medium replaced with buffer containing DCFDA (Abcam, Cambridge, UK) at 25 μM, and they were incubated at 37 °C for 45 min. After treatment with DCFDA, cells were washed and buffer replaced with medium containing the indicated combinations of hydrogen peroxide, Trolox, and NAC and treated for 3 h. Fluorescence was measured using an Infinite M1000 Pro with excitation wavelength of 485 nm and emission wavelength of 535 nm.

Dihydroethidium (DHE) assay was also used to evaluate intercellular ROS levels. After the transfection of HOXB13-V5, the cells were washed and culture medium replaced with buffer containing DHE (Abcam) at 5 µM for 30 min. After treatment with DHE, cells were washed and buffer replaced with medium containing the indicated combinations of PEG-catalase (Sigma-Aldrich Inc., St. Louis, MO, USA), antimycin A (Abcam), and NAC and treated for 3 h. Fluorescence was measured using an Infinite M1000 Pro with excitation wavelength of 500 nm and emission wavelength of 570 nm. Trolox directly scavenges superoxide and lipid radicals. NAC increases the intercellular cysteine levels and enhances glutathione synthesis, a major endogenous antioxidant. Thus, we used these compounds as antioxidants in the study.

### 2.7. Statistical Analysis

Statistical significance was analyzed using one-way ANOVA and Tukey’s post hoc test.

## 3. Results

### 3.1. HOXB13 Is Involved in the Cell Death Caused by Hydrogen Peroxide through Enhancement of Oxidative Stress

**Figure 1:**
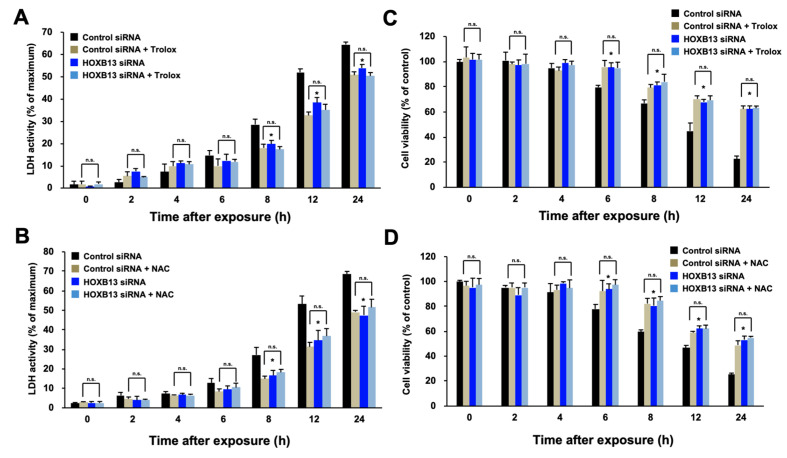
Effects of HOXB13 knockdown and antioxidants on cytotoxicity caused by hydrogen peroxide. A549 cells were transfected with control siRNA or HOXB13 siRNA for 24 h and then the cells were re-seeded onto 48-well plates and incubated for 24 h. Cells were exposed for the indicated period to hydrogen peroxide (400 μM) and Trolox (10 μM; **A**,**C**) or NAC (100 μM; **B**,**D**). Cell death was measured by LDH assay (**A**,**B**) or alamarBlue assay (**C**,**D**). All values are mean ± S.D. of three individual experiments. * *p* < 0.05 vs. Control siRNA. n.s. indicates not significant.

We previously reported that knockdown of HOXB13 in HEK293 cells reduced sensitivity to hydrogen peroxide; however, it was not clear if this acquisition of tolerance depended on a reduction in oxidative stress. To address this issue, we used human lung epithelial A549 cells because it is known that ROS production by harmful substances specifically contributes to damage in alveolar epithelial cells. We examined the sensitivity of HOXB13 knockdown cells to hydrogen peroxide with or without an antioxidant. Knockdown of HOXB13 decreased cytotoxicity caused by hydrogen peroxide in A549 cells; however, this suppression of cytotoxicity was not altered by further addition of NAC or Trolox that confers resistance to hydrogen peroxide (Figure 1A,B). We also evaluated cell death by alamarBlue assay, an assay for evaluating mitochondrial activity, and the results indicated same as the LDH assay (Figure 1C,D). These results indicate that knockdown of HOXB13 suppressed hydrogen peroxide induced cytotoxicity through the same pathway as the antioxidants. Thus, HOXB13 may be involved in enhancement of oxidative stress in cells.

### 3.2. Hydrogen Peroxide Increases HOXB13 Protein Levels via Increasing Its Transcription

**Figure 2:**
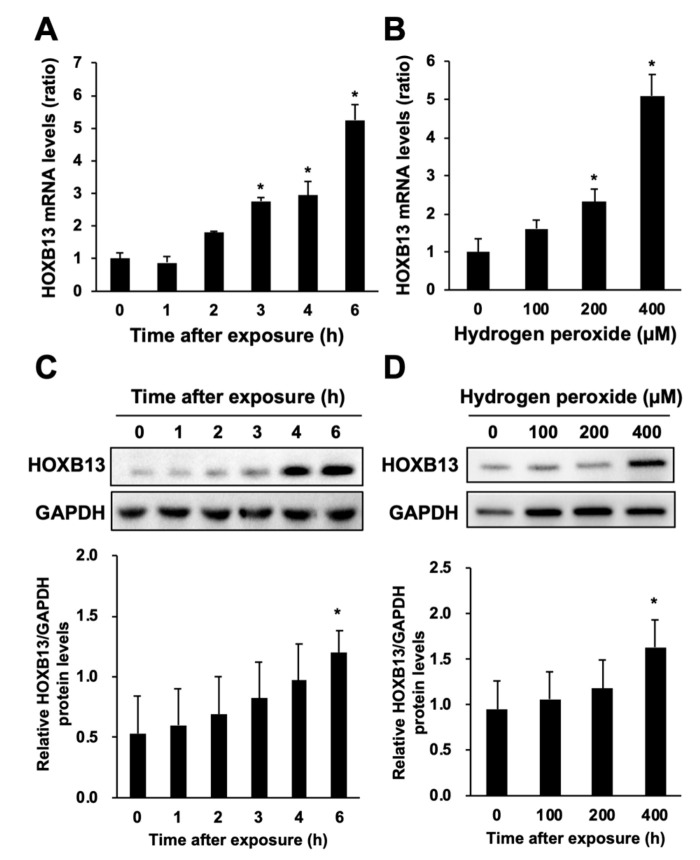
Effects of hydrogen peroxide on HOXB13 expression. A549 cells were seeded onto 6-well plates for 24 h. (**A**) Cells were exposed for the indicated period to hydrogen peroxide (400 μM) or (**B**) exposed to the indicated concentration of hydrogen peroxide for 6 h. The mRNA levels of HOXB13 and GAPDH were measured, and the relative values normalized to GAPDH are shown. (**C**,**D**) Upper panels indicate immunoblots for HOXB13 and GAPDH proteins under the same conditions as in **A**,**B**. Lower panels indicate densitometry analysis of the band intensity in **C**,**D**. All values are mean ± S.D. of three individual experiments. * *p* < 0.05 vs. 0 h or 0 μM.

**Figure 3:**
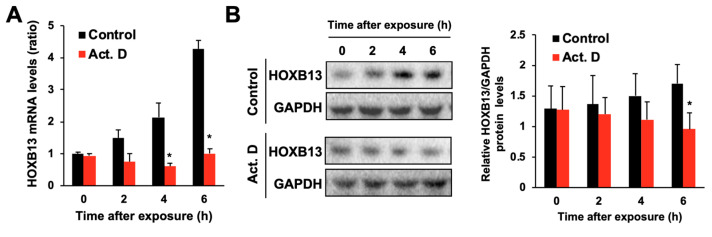
Effects of actinomycin D on the increase in the levels of HOXB13 expression by hydrogen peroxide. A549 cells were seeded onto 6-well plates for 24 h. Cells were exposed to hydrogen peroxide (400 μM) in the presence or absence of actinomycin D (Act. D; 1 μM). (**A**) The mRNA levels of HOXB13 and GAPDH were measured, and the relative values normalized to GAPDH are shown. (**B**) Left panel indicates immunoblots for HOXB13 and GAPDH proteins under the same conditions as in A. Right panel indicates densitometry analysis of the band intensity in the left immunoblot. All values are mean ± S.D. of three individual experiments. * *p* < 0.05 vs. control.

We examined the effect of hydrogen peroxide on the expression levels of HOXB13. The mRNA levels of HOXB13 were increased after hydrogen peroxide exposure in a time- and concentration-dependent manner (Figure 2A,B). The protein levels of HOXB13 were also increased by hydrogen peroxide treatment (Figure 2C,D). Conversely, treatment with Trolox and NAC had little effect on the expression levels of HOXB13 (data not shown). Thus, hydrogen peroxide upregulates the expression of HOXB13. To investigate the relationship between mRNA and protein levels of HOXB13 in the hydrogen peroxide-induced increase in its expression, we examined the expression variation of HOXB13 in the presence of actinomycin D (Act. D), a transcriptional inhibitor. In the presence of Act. D, there was little increase in the levels of HOXB13 mRNA and protein after hydrogen peroxide treatment (Figure 3). These results indicate that hydrogen peroxide increases HOXB13 protein levels by increasing its transcription.

### 3.3. Overexpression of HOXB13 Caused Cell Death via Endogenous ROS Production

**Figure 4:**
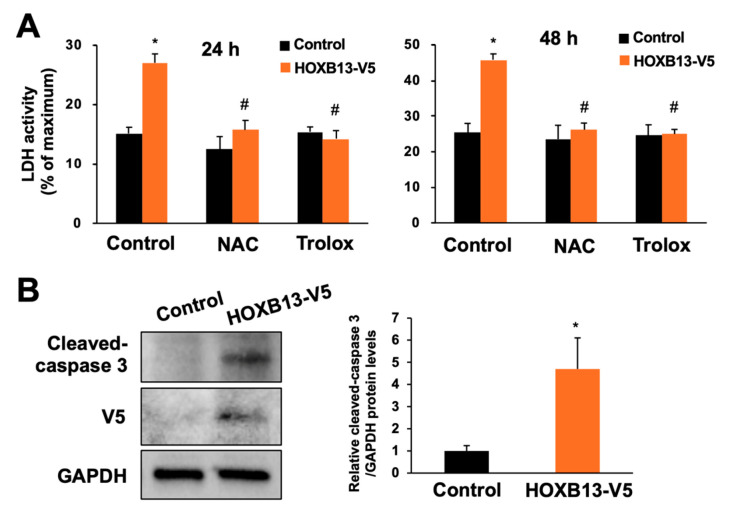
Effects of overexpression of HOXB13 on cell viability. A549 cells were transfected with control plasmid or HOXB13-V5-expressing plasmid for 24 h and then the cells were re-seeded onto 48-well plates. (**A**) After the re-seeding and culture for 24 h (left panel) or 48 h (right panel) with or without NAC (100 µM) or Trolox (10 µM), cell death was measured by LDH assay. (**B**) After the re-seeding and culture for 24 h, immunoblots for cleaved-caspase 3, V5-tag, and GAPDH were performed (left panel). Right panel indicates densitometry analysis of the band intensity in the left immunoblot. All values are mean ± S.D. of three individual experiments. * *p* < 0.05 vs. control, ^#^ *p* < 0.05 vs. control group that was transfected with the HOXB13-V5-expressing plasmid.

We next constructed a HOXB13-V5-expressing plasmid and transfected it into A549 cells to investigate whether induction of HOXB13 expression by hydrogen peroxide is involved in cytotoxicity. The overexpression of HOXB13-V5 under these conditions was confirmed by immunoblotting with an antibody recognizing the V5-tag. Cell viability was reduced by approximately 20% at 24 h and 40% at 48 h by HOXB13 overexpression compared with the control cells (Figure 4A). Most of the cells were dead within 72 h of transfection (data not shown). Furthermore, cell death due to HOXB13 overexpression was markedly inhibited by addition of NAC or Trolox (Figure 4A). These results suggest that ROS may be involved in the induction of cell death by HOXB13 overexpression in A549 cells. Increased ROS can often cause the induction of apoptosis; thus, we evaluated cleaved-caspase 3 levels as an apoptosis marker. HOXB13 overexpression slightly induced cleavage of caspase-3 (Figure 4B); this result indicates that HOXB13 could also contribute to apoptosis induction.

**Figure 5:**
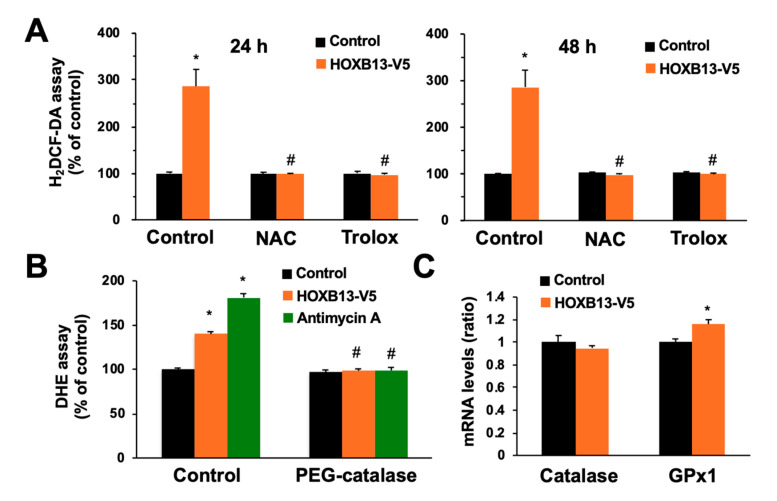
Effects of overexpression of HOXB13 on reactive oxygen species (ROS) production. A549 cells were transfected with control plasmid or HOXB13-V5-expressing plasmid for 24 h and then the cells were re-seeded onto 48-well plates. (**A**) After the re-seeding and culture for 24 h (left panel) or 48 h (right panel) with or without NAC (100 µM) or Trolox (10 µM), ROS levels were measured by DCFDA assay. (**B**) After the re-seeding and culture for 24 h with or without of antimycin A (150 µM) and/or PEG-catalase (1000 U/mL), ROS levels were measured by DHE assay. (**C**) After the re-seeding and culture for 24 h, the mRNA levels of catalase and GPx1 were measured and the relative values normalized to GAPDH are shown. All values are mean ± S.D. of three individual experiments. * *p* < 0.05 vs. control, ^#^ *p* < 0.05 vs. control group that was transfected with the HOXB13-V5-expressing plasmid.

Next, we examined the intracellular ROS levels by using the H2DCF-DA assay to investigate the involvement of ROS in cell death due to HOXB13 overexpression. HOXB13 overexpression significantly increased intracellular ROS levels, but this increase was largely absent upon treatment with NAC or Trolox (Figure 5A). Intracellular ROS levels were also examined by DHE assay as well and the results support the same conclusion (Figure 5B). In the same condition, we confirmed an increase in ROS levels by antimycin A as positive control (Figure 5B). Moreover, the increase in ROS levels by HOXB13 overexpression or antimycin A was completely canceled by addition of PEG-catalase, a membrane permeable hydrogen peroxide specific scavenger (Figure 5B). We also investigated the expression of factors involved in the removal of intracellular hydrogen peroxide using the qPCR, since HOXB13 is a transcription factor. HOXB13 overexpression did not decrease the mRNA levels of catalase and glutathione peroxidase 1 (GPx1) (Figure 5C). Taken together, hydrogen peroxide induces HOXB13 expression in cells; this may contribute to an increase in endogenous ROS production, which causes cell death.

## 4. Discussion

Our present results suggest that HOXB13 is upregulated by hydrogen peroxide and that an increase in its levels is associated with enhancement of oxidative stress, which is followed by cell death [16]. Although the detailed mechanisms underlying the above phenomena are obscure, the present study clearly indicates that HOXB13 may be a promising target factor to study cellular toxicity induced by various oxidative stress inducers in pulmonary cells.

Excessive oxidative stress is involved in damage to DNA, lipids, or protein and causes cell death [17,18]. Living cells protect themselves from oxidative stress-induced cytotoxicity through the action of cellular defense factors such as Nrf2 [19] or Hsf1 [20]. Contrary, less is known about the transcription factors involved in oxidative stress-induced cytotoxicity, e.g., EST1 is known to be involved in ROS production via inducing NADPH oxidase [21]. Our results suggest that HOXB13 may be a novel factor that is involved in enhancement of endogenous ROS production (Figure 4 and Figure 5). Endogenous ROS are mainly produced by mitochondria, NOX, nitric oxide synthase, and xanthine oxidase [22,23,24,25]; however, HOXB13 is located in the nucleus. Thus, HOXB13 may increase endogenous ROS via promoting transcription of the downstream genes associated with intracellular ROS production. Elucidation of the detailed mechanism through comprehensive screening such as DNA microarrays and RNA sequencing will clarify the new toxicological significance of HOXB13 as an endogenous oxidative stress inducer.

As described in the introduction, the induction of HOXB13 gene expression is known to be negatively regulated by a long non-coding RNA, HOXB13-AS [10]; however, transcriptional regulation of HOXB13 has not been fully elucidated. We examined the promoter activity of the HOXB13 gene and found that a region −250 to −500 bp upstream of the transcriptional start site was important for the transcriptional promotion of HOXB13 by hydrogen peroxide (unpublished observation). Furthermore, candidate transcription factors that could bind to this promoter region and promote HOXB13 transcription were identified as SP-1 and HIF-1 by using the Eukaryotic Promoter Database (data not shown; https://epd.epfl.ch//). Hydrogen peroxide has been reported to activate HIF-1 [26] and Sp1 [27]; thus, these factors may be involved in the induction of HOXB13 expression by hydrogen peroxide. However, further detailed studies are needed to reliably identify the transcription factors involved in the induction of HOXB13.

Although it has been reported that HOXB13 suppresses the cell cycle in some human prostate cancer cells in response to its increased expression [28], overexpression of HOXB13 has not been reported to cause cell death, unlike in the present study. Therefore, it is likely that HOXB13 causes cell death by promoting the induction of oxidative stress through an unknown mechanism. At least mRNA levels of catalase and glutathione peroxidase were largely unchanged by HOXB13 overexpression (Figure 5C); these indicate that decrease of antioxidant systems may not be involved in the enhancement of ROS production by HOXB13 overexpression. HOXB13-mediated ROS production was completely canceled by addition of PEG-catalase, a membrane permeable hydrogen peroxide specific scavenger (Figure 5B). These results suggested that HOXB13-mediated ROS production is mostly hydrogen peroxide, and this may be the cause of cell death. Furthermore, as mentioned above, this is the first study to suggest that HOXB13 functions as a novel factor involved in the induction of oxidative stress and will have a significant impact on future oxidative stress-related research. However, we have yet to find that HOXB13 levels are increased by hydrogen peroxide in any other cell types apart from HEK293 and A549 cells. In addition, high concentrations of hydrogen peroxide would cause secondary ROS production and imbalance cell redox status that is related to unexpected artificial cellular responses; thus, these are limitations of our study. In the future, similar studies on various cell types and primary cultured cells will be necessary, and, if possible, verification studies using experimental animals will also be required.
